# *Unfolding* the Unfolded Protein Response: Unique Insights into Brain Ischemia

**DOI:** 10.3390/ijms16047133

**Published:** 2015-03-30

**Authors:** Thomas H. Sanderson, Molly Gallaway, Rita Kumar

**Affiliations:** 1 Cardiovascular Research Institute and Department of Emergency Medicine, Wayne State University School of Medicine, Detroit, MI 48201, USA; E-Mail: tsanders@med.wayne.edu; 2 Department of Emergency Medicine, Wayne State University School of Medicine, Detroit, MI 48201, USA; E-Mail: molly.gallaway@gmail.com; 3 Cardiovascular Research Institute and Departments of Emergency Medicine and Physiology, Wayne State University School of Medicine, Detroit, MI 48201, USA

**Keywords:** cardiac arrest, brain ischemia, unfolded protein response, PKR-Like Endoplasmic Reticulum Kinase (PERK), Inositol Requiring Enzyme 1 (IRE1), Activating Transcription Factor 6 (ATF6), Glucose-Regulated Protein 78/Binding Protein (GRP78/BiP)

## Abstract

The endoplasmic reticulum (ER) is responsible for processing of proteins that are destined to be secreted, enclosed in a vesicle, or incorporated in the plasma membrane. Nascent peptides that enter the ER undergo a series of highly regulated processing steps to reach maturation as they transit the ER. Alterations in the intracellular environment that induce ER stress are thought to interrupt these processing steps, and result in unfolding of proteins in the ER. Accumulation of unfolded proteins concurrently activates three transmembrane stress sensors, IRE1, ATF6 and PERK, and is referred to as the Unfolded Protein Response (UPR). Our understanding of the mechanisms of UPR induction has been assembled primarily from experiments inducing ER stress with chemical and genetic manipulations. However, physiological stress often induces activation of ER stress sensors in a distinct manner from the canonical UPR. The unique activation profiles *in vivo* have prompted us to examine the mechanism of UPR activation in neurons following cerebral ischemia.

## 1. Introduction

Cardiac arrest continues to be leading causes of death and disability in the US. Although there has been significant advancement in finding innovative methods to restore pumping of blood throughout the body, little progress has been made in averting brain damage and often death from the initial ischemic event. The brain, as the central controller of human body, has evolved to rapidly respond to various stimuli. Specifically, neurons react to alterations in both incoming information and extracellular environment. Although there are a plethora of cellular changes in neurons following cerebral ischemia, one major organelle that responds to alterations in the environment is the endoplasmic reticulum (ER). This highly specialized organelle is by nature both physically and functionally distinct from the surrounding cytoplasm, however, is impacted by shifts in the neuron and the extracellular environment. 

Typically, the ER milieu serves to facilitate the proper processing of a distinct set of proteins destined to be secreted from the cell, integrated into membranes, or compartmentalized in other organelles. The role of the ER is not limited to the processing of proteins; instead in smooth ER, the lumen provides a suitable environment for the synthesis of steroids and lipids, and in sarcoplasmic ER, the primary luminal function is calcium sequestration. To sustain proper function, the ER must maintain an oxidizing environment, elevated Ca^2+^ enriched with specific molecular chaperones, isomerases, and glycosylation enzymes that all aid in polypeptides achieving final functional conformation [1]. 

Stressful stimuli alter the internal milieu of the ER, which results in errors in ER-protein processing [2]. Regulatory mechanisms within the ER are then activated to ensure that homeostasis is restored following the stressful event. This cellular response to ER stress has been a highly researched topic with implications in multiple pathologies, including cerebral ischemia. For over 30 years, researchers have expanded our understanding of the ER stress response termed the “Unfolded Protein Response” (UPR). Here, we review the UPR and its involvement in cerebral ischemia. 

## 2. The Unfolded Protein Response (UPR) 

Our understanding of the ER stress response has expanded greatly over the last three decades. We now know that accumulation of misfolded proteins in the ER can elicit an array of cellular responses including: (1) reduction or inhibition in the rate of protein synthesis [2]; (2) upregulation of genes that encode for ER chaperones, enzymes, and structural components of the ER, enabling the organelle to process more proteins [3]; and (3) initiation of programmed cell death (apoptosis) if the stress is too severe [2,4]. These UPR cellular responses are downstream from parallel activation of three key ER-transmembrane proteins: IRE1, ATF6, and PERK. 

### 2.1. Inositol Requiring Enzyme 1 (IRE1)

The first ER stress sentinel to be identified was IRE1 (Inositol-Requiring Enzyme), initially discovered in yeast [5]. IRE1 is an ER-transmembrane protein that is normally present in its monomeric form. Upon stress to the ER, IRE1 monomers homoligomerize and trans-autophosphorylate to become an active endoribonuclease [6]. Active IRE1 complexes cleave *Hac1* mRNA in yeast, which is then ligated by the tRNA ligase Trl1 [7]. The cleaved *Hac1p* is translated into a transcription factor that regulates transcription of UPRE (unfolded protein response element) containing promoters that are necessary for ER resident protein transcription [7]. The mammalian equivalent proteins, IRE1α and IRE1β, were found to act functionally similar to IRE1 in yeast [8]. Moreover, the IRE1 signaling pathway is conserved with active IRE1 endoribonuclease activity processing *XBP-1* mRNA, ligated by an unknown enzyme in higher eukaryotes, and subsequent translation into a transcription factor that regulates genes containing ERSE promoters (ER stress response element) [5]. 

In addition to initiating transcriptional activity in the nucleus, mammalian IRE1 activates the JNK (Jun *N*-terminal kinase) signaling pathway and interacts with cell death machinery, such as caspase-12. The JNK pathway plays an unknown role in the regulation of cell survival and apoptosis. Although IRE1 can activate apoptotic related pathways, activation of IRE1 is generally thought to enhance cell survival [7]. 

### 2.2. Activating Transcription Factor 6 (ATF6)

Next, ATF6 (Activating Transcription Factor 6) is concomitantly activated upon stress to the ER. ATF6 is an ER-transmembrane protein that is activated via translocation from the ER to the Golgi and proteolysis by S1P (site 1 protease) then S2P (site 2 protease) [5]. Following cleavage, ATF6-p50 (a cytosolic DNA-binding portion fragment) is released and translocates to the nucleus where it binds to endoplasmic reticulum stress elements (ERSEs) to amplify transcription of the UPR genes [7]. In addition to its role as a transcription factor, ATF6 targets processed XBP-1 to increase binding of XBP-1 to ERSE and enhance transcription of genes needed for the UPR response [9], thus suggested a pro-survival role for ATF6. However, ATF6 processing has also been implicated in cell death [10].

### 2.3. PKR-Like Endoplasmic Reticulum Kinase (PERK)

The final ER stress sensor protein that was discovered is the ER-transmembrane PERK (Pancreatic ER kinase or PKR-like ER kinase), which is responsible for attenuating protein translation. Upon stress to the ER, PERK monomers homoligomerize and trans-autophosphorylate to become an active eIF2α kinase [4,6,7,11]. Phosphorylation of the α subunit of the eukaryotic initiation factor 2 (eIF2α) is responsible for impeding the global rate of protein synthesis [12,13]. In addition to PERK phosphorylating eIF2α and decreasing protein translation, PERK activation has downstream effects. Particular mRNAs, including ATF4, are synthesized due to the increase in eIF2α(P) via PERK. Notably, ATF4 increases the transcription of a proapoptotic factor, C/EBP homologous protein (CHOP).

### 2.4. Evolution of the UPR

The initial discovery of homeostatic regulation within the ER has led to identification of many of the signaling components involved in downstream progress of UPR. Sambrook pioneered the UPR field with his report on the response of mammalian cells infected with a malfolded form of an ER-targeted viral protein. Accumulation of this aberrant ER protein resulted in upregulation of two proteins, GRP78 and GRP94 [14]. Prior to this discovery, it was postulated that underglycosylation of proteins increased GRP expression [15]. In fact, work by Pastan showed that the inhibition of glycosylation, basically a result of glucose starvation, resulted in increases in 78,000 and 95,000 Da “glucose related proteins” [16,17]. These pioneering experiments provided founding evidence that cells possess a highly integrated response system that is activated by stressful stimuli within the ER, most notably the accumulation of aberrant unfolded proteins. Thus, these investigators came up with the intuitive conclusion that a build-up of malfolded proteins in the ER lumen, as a result of stress to the ER, triggered the UPR. These pivotal studies demonstrated that accumulation of malfolded proteins led to up-regulation of the chaperone proteins GRP78 and GRP94, which laid the foundation for the rapidly growing discipline of ER stress and UPR. In fact, to date many investigators correlate the up-regulation of GRP78 and GRP94 as evidence of the UPR [18,19,20]. 

### 2.5. The Role of GRP78 (BiP)

Although the downstream pathways of all three UPR sensor proteins affect the transcription and translation of genes that enhance the processing capacity of the ER, it is GRP78/BiP that is proposed to be the master initiator of the UPR. Moreover, it is the depletion of GRP78 that results in widespread activation of the UPR. Depletion of GRP94 activates expression of select genes, distinct from a typical ER stress response [21]. 

GRP78/BiP is an ER molecular chaperone that was termed a Glucose Regulated Protein (GRP) because the increase in expression of GRPs (GRP78 and GRP94) was initially identified following glucose starvation [16,17]. It was later discovered that glucose starvation impairs protein glycosylation, which disrupts the correct folding of proteins within the ER. The accumulation of misfolded proteins in the lumen ultimately led to an increase in expression of GRP78 and GRP94 [14,15]. 

GRP78 is known to be expressed constitutively and bind to the luminal domains of PERK, IRE1, and ATF6 [22]. Under conditions that promote accumulation of misfolded proteins, GRP78 is proposed to dissociate from the ER stress sensors and bind hydrophobic regions of unfolded proteins [22,23,24]. In support of this theory, using mutation experiments involving the luminal end of IRE1, Chapman *et al.* [1] observed that GRP78/BiP could prevent IRE1 oligomerization by binding to the luminal domain of IRE1. Mutations in the IRE1 luminal domain that prohibited binding of GRP78/BiP to IRE1 resulted in IRE1 activation. The disassociation of GRP78/BiP from IRE1 was speculated to result from an increase in concentration of misfolded proteins sequestrating GRP78/BiP. Thus the link of GRP78 disassociation from the luminal end of IRE1 to bind unfolded proteins following disturbances in the ER remained. Moreover, since GRP78/BiP was shown to bind the luminal end of PERK along with the lack of alternative activation mechanisms, this mechanism of activation involving disassociation of GRP78/BiP to bind unfolded proteins was the template of activation for all three ER sentinels. Recent studies have expanded on this mechanism and have demonstrated that in yeast, hydrophobic domains of unfolded proteins can directly bind IRE-1, suggesting the mechanism of UPR sensor activation by GRP78/BiP dissociation may be more diverse that originally postulated [25]. 

## 3. The UPR in Cerebral Ischemia

**Figure 1:**
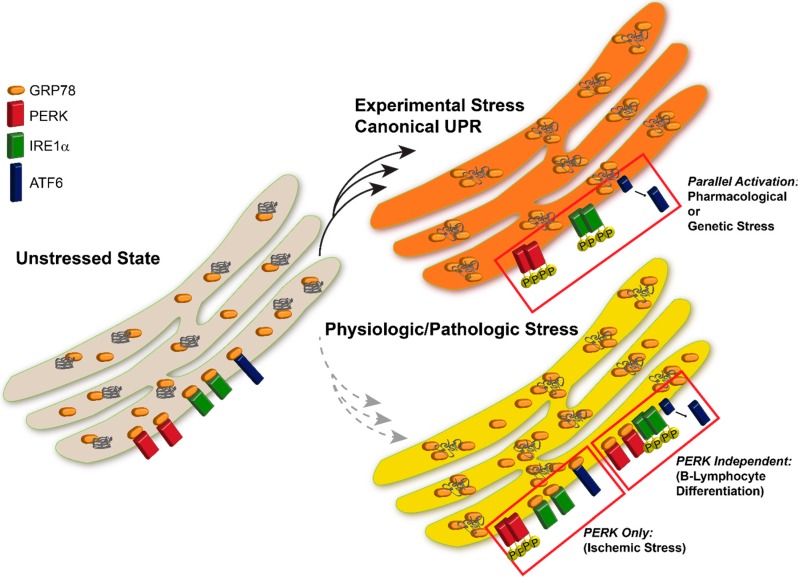
UPR Activation in Following Experimental *vs.* Pathological Stress. Induction of the UPR with pharmacologic agents results in parallel activation of all three UPR sensor proteins. Physiologic or pathological stresses often induce activation of specific components of the UPR, however all sensors are not activated uniformly following all insults.

Following global brain ischemia, an atypical UPR response is observed. In Kumar *et al.* [26] we observed that inhibition of protein synthesis in select regions of the brain was due to activation of PERK. PERK activation suggested that the UPR was triggered during cerebral ischemia, a reasonable theory considering the ion flux necessary for maintaining membrane potential and REDOX shifts where ATP-dependent. After examining the PERK, IRE1, and ATF6 pathways in the brain stem, cerebral cortex, and hippocampus following 10 min global brain ischemia and four hours reperfusion in rats, we observed PERK activation and downstream eIF2α phosphorylation leading to inhibition of protein translation but no increase in CHOP or ATF4, the downstream components of PERK signaling. Moreover, IRE1 and ATF6 were not activated, which were surprising results with regards to the current UPR paradigm and ER stress. Of the three UPR-stress sensor proteins, only PERK was activated (Figure 1) [26,27]. The atypical activation of the UPR components was an interesting finding due to the prior belief that all three components of the UPR act simultaneously. Additionally, our group discovered that PERK activation and subsequent phosphorylation of eIF2α was independent of nascent protein load in the ER, suggesting that PERK was activated by a novel mechanism [27]. 

In yet another example of unfolded protein-independent activation of PERK, Gomez *et al.* [27] showed that depletion of ATP in pancreatic β-cells results in activation of only PERK, independent of both unfolded proteins in the ER and IRE1 activation, similar to the findings in transient global brain ischemia. These findings suggest that PERK can also be activated by other cellular disturbances independent of unfolded proteins and that unfolded proteins may be sufficient but not necessary to activate PERK. 

The current paradigm is that the UPR is a stress response elicited by the cell to restore homeostasis to the ER by restoring proper polypeptide processing. During cerebral ischemia, the lack of the canonical UPR may destine the cell to death because of the lack of transcriptional and translational response afforded when IRE and ATF6 are activated. 

## 4. Limitations of Our Current Models of the UPR

Studies identifying the various components of the UPR were primarily conducted in cell culture and then translated to *in vivo* models. Although these models are extremely useful in elucidating ER stress signal transduction, a limitation of chemically inducing ER stress is that the degree of ER stress is not physiologically relevant and therefore may lead to specific results. The initial studies of the UPR were in cells transfected with ER localized proteins incapable of folding. These studies of aberrant ER protein established the idea that the UPR is triggered by unfolded proteins. In subsequent studies, when ER stress was induced pharmacologically, the concomitant activation of all three UPR sensors IRE1, ATF6, and PERK promoted the idea that a single mechanism of activation for all three mammalian UPR sensors exists, *i.e.*, accumulation of unfolded proteins causes dissociation of GRP78 from the luminal end of UPR sensor proteins, resulting in their activation. While cell culture, genetic manipulation and pharmacologic induction of the UPR were valuable and necessary tools to elucidate cellular mechanisms, they must be recognized as experimental situations. These chemicals induce severe ER stress in cell culture and initiate a multitude of other changes in the ER including alterations in protein processing, post-translational modifications, signal transduction and protein binding, which renders it difficult to decipher the precise stimuli that activate the UPR *in vivo*. The acute experimental conditions that are used to elucidate and understand cellular signaling mechanisms may not represent what is occurring in a physiological setting *in vivo* or mimic what occurs during the pathophysiology of disease. 

Although Sambrook’s experiments demonstrated that unfolded proteins are sufficient to activate the UPR, the inverse experiment was never conducted. Thus, by basing our understanding on the initial experimental model where a large increase in unfolded proteins in the ER lumen elicited the UPR, we may be limiting our understanding of ER regulatory mechanisms. The UPR field has continued to evolve from the initial studies where unfolded proteins were sufficient to active the UPR, however, none have shown whether unfolded proteins were necessary to elicit this ER stress response. Albeit, GRP78/BiP repression of ER-stress-signal transducer activity has been demonstrated as the key determinant of UPR activation, the dependence of this process on accumulation of unfolded proteins has not been demonstrated [22,28]. One possible explanation of activation of ER stress sensor proteins is conformational changes of their luminal domains as a result of stress-induced alterations in the ER milieu. In fact, it has been postulated that unfolded proteins directly interact with a groove on the ER-stress signal transducers IRE1 and PERK to promote a conformational change in the luminal domains [7,29]. This scenario would result in GRP78 dissociation in contrast to sequestration of GRP78 directly by unfolded proteins. Although unfolded proteins may be one of many perturbations that can affect GRP78 binding, others factors such as changes in the inorganic milieu of the ER (*i.e.*, Ca^2+^, redox, ATP), or stress sensing co-factors (Erdj5) could promote conformational changes in the luminal domain of the three ER-stress transducers and alter the binding of GRP78 [30,31].

## 5. Other Non-Canonical UPR Situations

In UPR related diseases, there is added complexity where multiple ER stress initiating stimuli are present. In disease states, loss of Ca^+2^ in the ER, inhibition of transport to the Golgi from the ER, altered redox environment, ATP depletion, overproduction of proteins, and protein degradation inhibition effects the protein processing of the ER. If the current notion based on unfolded proteins being the common stimuli that leads to activation of all three UPR sensors was universal, we would expect activation of all three ER stress-sensor proteins in all circumstances. However, it has been identified that certain cell types undergo a non-canonical UPR [32,33,34]. 

Ma *et al.* [33] discovered that the PERK branch of the UPR is *dormant* during plasma cell differentiation. Following plasma cell differentiation, immunoglobin (Ig) heavy and light chain synthesis increases to significantly boost the production of antibodies [35]. In order to assess the relationship between the differentiation process and the UPR, a B-lymphocyte line was induced using both standard stressors such as thapsigargin (depletes Ca^+2^ from the ER) or tunicamycin (blocks protein glycosylation in the ER) and LPS (lipopolysaccharide)-induced differentiation [33]. Using LPS is a method to study the B-lymphocyte differentiation process *in vitro* [35]. All of the branches were activated with the standard stressors, but the canonical UPR response was faulty in the LPS-induced differentiation of the B-lymphocytes. PERK activation was obscure and none of the downstream targets of PERK where activated. Thus it was concluded that plasma cell differentiation attenuates the PERK branch of the UPR (Figure 1) [33].

Once B-lymphocytes are stimulated, they differentiate into antibody secreting cells (ASC). During stimulation, CHOP is temporarily produced and ASCs go on to produce a large amount of Ig and then typically undergo apoptosis [33]. Masciarelli *et al.* [34] showed that while CHOP does not affect the ASC lifespan or differentiation, CHOP−/−ASCs showed a decrease in IgM secretion. Hence, it is hypothesized that CHOP is necessary for proper IgM folding and for quality control purposes. Additionally, the finding that CHOP−/−ASCs had increased sensitivity to thapsigargin and tunicamycin goes against previous findings that CHOP expression is related to a maladaptive UPR response [34]. For example, CHOP is correlated with a maladaptive stress response in Schwann cells [36]. Thus, the cell-specific roles of CHOP further support the notion that components of the UPR can be differentially regulated.

A recent study by Lin *et al.* [4] demonstrated the divergent roles of IRE1 and PERK activation, while PERK activation lead to cell death, activation of IRE1 promoted cell survival. Examples of these divergent effects suggest a physiologic need for the cell to have independent regulatory mechanisms to control each arm of the UPR.

It still remains unclear what exactly is causing PERK activation in pathological and physiological systems, but parallel investigations into how IRE1 is activated directly with unfolded proteins are being conducted and may provide insight into the mechanism of PERK activation [29,36]. These findings of selective inhibition of branches of the UPR introduce the idea that activation of each branch of the UPR might be further regulated to respond independently of the others as both a necessary and valuable tool for different physiological processes in various cells and tissues.

## 6. Conclusions

The UPR is activated when a cell undergoes ER stress through activation three major stress sensors, IRE1, ATF6, and PERK. According to the current dogma of the UPR, ER stress sensors are activated via unfolded proteins promoting disassociation of GRP78 from the sensors’ luminal domains, thus triggering the parallel activation of the three stress transducers. Although the UPR stress sensor activation has been found to correlate with increased GRP78 expression, it has not been determined whether their activation is directly due to unfolded proteins or instead, direct or indirect dissociation of GRP78. Moreover, it has been found that stimuli other than unfolded proteins, such as Ca^2+^, redox, or ATP, can also regulate GRP78 binding, to activate the UPR [30,31]. Studies have shown that the UPR components can be differentially regulated and activated independently. In comparison to the canonical UPR elicited via standard drugs, such as tunicamycin, thapsigargin, or DTT, ailment to the human body involves a number of complex biological processes that may result in a more tailored and specific activation of the individual arms of the UPR. This ability to individually regulate the UPR stress sensors would be advantageous and a key factor for maintaining cellular homeostasis in response to diverse physiological events. As the brain has evolved, neurons in specific regions have needed to modify cell signaling to accommodate precise functions, which could alter an ER stress response. Finally, we must be cautious to presume execution of the full UPR in pathological conditions following expression of select proteins. Further research must be done to determine the exact mechanism(s) that may activate IRE1, ATF6, and/or PERK and the role these mechanisms play in health and disease.
